# Multi-Country Evaluation of Affective Experience: Validation of an Abbreviated Version of the Day Reconstruction Method in Seven Countries

**DOI:** 10.1371/journal.pone.0061534

**Published:** 2013-04-23

**Authors:** José Luis Ayuso-Mateos, Marta Miret, Francisco Félix Caballero, Beatriz Olaya, Josep Maria Haro, Paul Kowal, Somnath Chatterji

**Affiliations:** 1 Instituto de Salud Carlos III, Centro de Investigación Biomédica en Red de Salud Mental, CIBERSAM, Madrid, Spain; 2 Department of Psychiatry, Hospital Universitario de La Princesa, Instituto de Investigación Sanitaria Princesa (IP), Madrid, Spain; 3 Department of Psychiatry, Universidad Autónoma de Madrid, Madrid, Spain; 4 Parc Sanitari Sant Joan de Déu, Universitat de Barcelona, Sant Boi de Llobregat, Barcelona, Spain; 5 Department of Health Statistics and Information Systems, World Health Organization, Geneva, Switzerland; 6 University of Newcastle Research Centre on Gender, Health and Ageing, Newcastle, New South Wales, Australia; Federal University of Rio de Janeiro, Brazil

## Abstract

**Background:**

The Day Reconstruction Method (DRM) was developed to assess affective states as measures of experienced well-being. The present study aimed to validate an abbreviated version of the DRM in a representative sample of the population in seven countries (China, Ghana, India, Mexico, Russia, South Africa, and Spain), and to examine whether there are country differences in affect and in the relationships among the activities based on the similarity of the affect associated with each of them.

**Methods:**

Interviews were conducted with 47,222 non-institutionalized adults from seven countries, using an abbreviated version of the DRM. A cluster analysis was carried out to classify activities on the basis of the similarity of the associated affect. In each country, the factorial structure of the affect adjectives was tested through Confirmatory Factor Analysis. Internal consistency and construct validity were also assessed. Moreover, the differences in affect across countries and the diurnal cycles of affect were evaluated.

**Results:**

The DRM showed adequate psychometric properties regarding reliability and construct validity in all countries. Respondents from Ghana and South Africa reported more positive net affect whereas Indian respondents reported less positive net affect. Most of the countries showed a similar diurnal variation of affect, which tended to improve throughout the day.

**Conclusions:**

The results show that this abbreviated version of the DRM is a useful tool for multi-country evaluation of experienced well-being.

## Introduction

The Day Reconstruction Method (DRM) [1] is a tool for studying well-being which assesses how people spend their time, with whom they spend time and how they describe the experiences associated with the activities and settings of their lives. The DRM may have multiple applications, from evaluating the impact of different illnesses, or analyzing social and economic stressors to evaluating policies and measuring the well-being of the society [1].

Kahneman *et al.* [1] administered the first version of the DRM to 909 working women in Texas in the United States of America. Respondents were asked to first think about the previous day by writing down a diary consisting of a sequence of episodes covering the entire waking day. They then described each episode by answering questions about each episode and associated feelings they experienced.

This original self-administered paper-and-pencil version of the DRM takes 45–75 minutes to complete [1], [2]. Therefore, it has been used mainly with small, convenience samples from higher income countries [3]–[5], particularly the United States [2], [6], [7].

Due to time and economic constraints, most national comparisons of well-being assess evaluative well-being based on global life satisfaction or general happiness questions [8]. However, cultural comparisons based on these self-reports are difficult to interpret, and have some important sources of bias, e.g. memory bias and positivity bias [9].

Experienced well-being is perhaps a more challenging construct to measure. In recent years, some attempts have been made to evaluate experienced well-being in larger samples. Krueger and Stone [10] designed a telephone survey based on the American Time Use Survey and the DRM: the Princeton Affect and Time Survey (PATS). This approach is a good approximation of the gold standard, Experience Sampling Method. The Gallup World Poll [11] was able to obtain information about well-being from nationally representative samples, with questions about whether respondents experienced certain positive and negative feelings a lot during the previous day. While providing valuable insights, the time use and associated affective experience are not collected for each activity limiting its usefulness in fully characterizing experienced well-being.

A brief measure of experienced well-being that could be used in large national representative survey samples could overcome the logistical barriers needed to generate comparable data on well-being across countries worldwide. One example of an abbreviated version of the DRM that can be used in large population surveys, irrespective of literacy levels and the ubiquity of telephones, has been recently validated in Jodhpur, India as part of the World Health Organization's Study on Global Ageing and Adult Health (SAGE) [12].

The present study aimed to validate this abbreviated version of the DRM in a representative sample of the population of seven countries, and to examine whether country differences exist in affect and in the activity-affect relationships.

## Materials and Methods

### Sample and Procedure

The data were obtained from SAGE and the Collaborative Research on Ageing in Europe (COURAGE in Europe), which are two multi-country projects compiling comprehensive data on the health and well-being of adult populations and the ageing process. Both studies collected data on respondents aged 18+ years, with an emphasis on populations aged 50+ years, from nationally representative samples. The data from Spain presented in the present paper come from COURAGE in Europe, whereas the data from the other six countries come from SAGE. The details of the design and methods for SAGE are published elsewhere [13].

The sample consists of 47,222 non-institutionalized adults aged 18-plus years from China (14,811), Ghana (5108), India (11,230), Mexico (2742), Russia (4355), South Africa (4223), and Spain (4753). Face-to-face interviews were conducted at the respondents' homes by trained interviewers. The individual response rate ranged from 53% in Mexico to 93% in China.

### Measures

Respondents were also asked to provide demographic information (age, sex, education level, marital status, residential location, work status) at the beginning of the interview. The abbreviated version of the Day Reconstruction Method [1] (available at http://www.who.int/healthinfo/systems/sage/en/index.html) used to obtain information about participants' daily activities and their subjective well-being, was limited to a maximum of 15 minutes of interview time. Respondents were asked to reconstruct a portion (morning, afternoon, or evening) of their previous day's activities, and reported the positive and negative emotions associated with each activity. The data provided a picture of the participants' daily lives, including what they did, for how long, and who they were with, as well as a way of calculating how much of their time was spent feeling pleasant or unpleasant emotions.

Respondents were randomly assigned to complete one of the four different versions of the abbreviated DRM (sets A, B, C, and D). In sets A, B and C, respondents reconstructed only a portion of their previous day's activities (starting with morning, afternoon, or evening respectively) and responded to questions about each episode, including the nature of the activity (for example, working, shopping), any people who were present (for example, alone, with spouse), and the extent to which they experienced various feelings–worried, rushed, irritated or angry, depressed, tense or stressed, calm or relaxed, and enjoying on a 3-point response scale (1 = not at all, 2 = a little, and 3 = very much). In the sample from Spain, the scale ranged from 0 (not at all) to 6 (very much) with the remaining points unlabeled. In set D, participants reported the activities, people present, and feelings for each part of the day (morning, afternoon and evening) altogether, instead of activity by activity along with the respective accompanying emotion. In sets A, B and C, the day was recorded in an event-by-event manner, and the participants reported the time at which the first activity registered started and the duration of each activity. This information was used to estimate the affective state at each hour of the day. Set D recorded broadly what was done in the morning, afternoon, and evening, and therefore the duration of each activity was not reported. Set D was not used in Spain.

Two measures were calculated based on the scores obtained with the seven affect items: net affect and U-index [14]. Net affect was defined as the average of the two positive emotions (calm/relaxed and enjoying) minus the average of the five negative ones (worried, rushed, irritated/angry, depressed, and tense/stressed), that is, positive affect minus negative affect. For sets A, B and C, scores were weighted by activity duration. For set D, a raw score was calculated because the affect items were not associated with single activities. For comparisons between the seven study countries, net affect scores were expressed on a percentile scale, in which 100 represents the best affective state. Positive and negative affect were also expressed on the percentile scale. The U-index was obtained by calculating, for each participant, the proportion of time in which the highest-rated feeling was a negative one. In set D, the U-index was not calculated because the duration of each activity was not collected.

The questions were translated from English into the local languages, following the WHO translation guidelines for assessment instruments. This included a forward translation, a targeted back-translation, review by a bilingual expert group and the elaboration of a detailed translation report. Ethical approvals from the following institutions were obtained: Ethics Review Committee, World Health Organization; Ethics Review Committee, Parc Sanitari Sant Joan de Déu, Barcelona, Spain; Ethics Review Committee, La Princesa University Hospital, Madrid, Spain; Ethical Committee, Ghana Medical School, Accra, Ghana; Ethics Committee, OPM (School of Preventive and Social Medicine), Russian Academy of Medical Sciences, Moscow, Russia; Ethics Committee, Shanghai Municipal Centre for Disease Control and Prevention, Shanghai, China; Institutional Review Board, International Institute of Population Sciences, Mumbai, India; Research Ethics Committee, Human Sciences Research Council, Pretoria, South Africa; and Ethics Committee, National Institute of Public Health (INSP), Cuernavaca, Mexico. Written informed consent from each participant was also obtained.

### Statistical methods

The sociodemographic characteristics of respondents were recorded for each country. Differences in sociodemographic characteristics between the final and the excluded sample were assessed by means of unpaired *t*-tests (continuous variables) and χ^2^ tests (categorical variables).

Descriptive analyses generated mean scores of positive and negative adjectives (worried, rushed, irritated/angry, depressed, tense/stressed, calm/relaxed, and enjoying) for the different activities. Based on these means, a cluster analysis was carried out in order to identify the relationships among the activities coded in the DRM in each country, according to the similarity of the activity-affect relationship. Variables were standardized, and the Euclidean distance was employed as a dissimilarity measure. Divisive hierarchical clustering method was used. The average linkage method was chosen because it maximizes the cophenetic correlation coefficient (CCC) [15], which is a measure of how faithfully a dendrogram maintains the original pairwise distances. A bootstrap version of cluster analysis that evaluated how consistently the same clusters appeared over 10,000 runs was performed with a sub-sampled dataset. For this purpose, the pvclust [16] package for statistical software R was used. For each cluster in hierarchical clustering, *p*-values were calculated. The *p*-value of a cluster is a value between 0 and 1, which indicates how strong the cluster is supported by the data. The pvclust package provides two types of *p*-values: Approximately Unbiased (AU) *p*-value and Bootstrap Probability (BP) value before statistical adjustments were reported. AU *p*-value, which is computed by multiscale bootstrap resampling, is a better approximation to unbiased *p*-value than the BP value computed by normal bootstrap resampling. Clusters strongly supported by the data (with AU higher than 95%) were highlighted by rectangles.

In each country, the factorial structure (negative versus positive items) of the seven adjectives was tested through Confirmatory Factor Analysis (CFA) for categorical outcomes, with the robust weighted least square estimator (WLSMV; which does not have assumptions of multivariate normality), and using polychoric correlations [17] for categorical variables. Goodness-of-fit of the model with two latent factors and seven observable indicators was assessed according to standard recommendations [18]. Several indices were used to assess fit according to the values proposed in the literature for Structural Equation Modeling (SEM) with categorical outcomes [19]–[22]: a) lack of significance of χ^2^; b) comparative fit index (CFI)>0.95; c) Tucker-Lewis index (TLI)>0.95; d) root mean square error of approximation (RMSEA)<0.08; and e) weighted root mean square residual (WRMR)<1.0. Since the χ^2^ statistic is sensitive to sample size [23], the χ^2^ values might be inflated (and statistically significant) due to the large size of the sample, which might erroneously imply a poor data-to-model fit [24]. Burnham and Anderson [25] noted that model goodness-of-fit based on statistical tests becomes irrelevant with large sample sizes. Moreover, WRMR is size-dependant and can be unreliable when the sample has more than 2000 cases. Due to these considerations and the large size of the sample used, only CFI, TLI and RMSEA are reported in this study. One of the main advantages of RMSEA is that it allows the calculation of a confidence interval around its value [26]. Ninety percent confidence intervals for RMSEA are also reported.

In sets A, B and C, the affective state associated with each activity was coded. Because the affective state was not reported for each activity in set D, cluster analysis and CFA were carried out only with the activities reported in sets A, B and C, pooling the data corresponding to these sets.

Reliability was assessed in terms of internal consistency using the Raykov & Marcoulides [27] method for reliability evaluation with categorical items, by means of Mplus option for maximum likelihood estimation with robust standard errors (MLR). Composite reliability was estimated for each of the factors obtained after CFA. In set D, similar analyses were carried out to assess construct validity and reliability. In this case, the analyses were run by countries, pooling the responses for the morning, afternoon and evening questions.

Diurnal variation of affect was assessed in each country, using the affective state reported by respondents in each hour of the day. This information was calculated for each participant assigned to sets A, B, and C based on the time at which the first activity reported began and the duration of each activity. Cross-country differences in net affect and U-index were tested by an ANOVA test. Bonferroni tests for post-hoc pairwise comparisons were used. 95% confidence levels were considered in hypothesis tests. Since statistical significance of differences could be due to the large sample size, effect size measures (Cramer's *V* for contingency table chi-square tests, Hedges' *g* for unpaired *t*-tests and pairwise comparisons, and Cohen's *f* for ANOVA tests) are reported. Cohen's guidelines were used as a reference [28]: Hedges' *g* values of 0.20, 0.50, and 0.80 constitute small, medium, and large effect sizes, respectively; these values are, respectively, 0.10, 0.25, and 0.40, in case of Cohen's *f*; and 0.10, 0.30, and 0.50, in case of Cramer's *V* for chi-square test for 2×2 contingency tables. Cluster analyses were carried out using R version 2.10.1 [29]. Mplus version 6 [30] was employed for factor analysis modeling. The rest of the analyses were performed using Stata SE version 11 [31].

## Results

A total of 47,222 people from China, Ghana, India, Mexico, the Russian Federation, South Africa, and Spain were interviewed. However, 1564 (3.3% of the initial cases) were removed from these analyses because they did not answer the subjective well-being section analyzed in the present article. The respondents excluded from the survey did not differ (the differences were not significant or had a very small effect size) by sex (56.7% women in the final sample vs. 60.0% women in the sample removed, *p* = 0.001, Cramer's *V* = 0.02), mean age (58.06 (s.d. = 14.84) vs. 59.43 (s.d. = 17.74), *p*<0.001, Hedges' *g* = 0.09), percentage of people married or in partnership (71.1% vs. 69.2%, *p* = 0.030, Cramer's *V* = 0.01), or percentage of people currently working (41.9% vs. 40.9%, *p* = 0.409).

Table 1 presents the main characteristics of the 45,658 respondents included in this study. The percentage of these participants who completed each of the four sets was approximately 25% (10,250 in set A, 10,355 in set B, 10,123 in set C, and 10,347 in set D) in SAGE. In the sample from Spain, 1536 participants completed set A, 1507 set B, and 1540 set C. Significant differences in sociodemographic characteristics were found between countries, with effect sizes ranging from moderate to large. Higher differences were found for mean age and residential setting, with India presenting the lowest mean age and the highest percentage of population living in a rural setting.

**Table 1:** Demographic characteristics of respondents by country.

	China	Ghana	India	Mexico	Russia	South Africa	Spain	Effect size*
	*n* = 14 244	*n* = 4909	*n* = 11 205	*n* = 2629	*n* = 4209	*n* = 3879	*n* = 4583	
Female: *n* (%)	7625 (53.5)	2323 (47.3)	6868 (61.3)	1625 (61.8)	2715 (64.5)	2229 (57.5)	2505 (54.7)	0.10
Age, years: mean (s.d.)	60.32 (11.85)	60.11 (14.09)	50.02 (16.60)	63.00 (14.30)	62.36 (13.02)	60.36 (12.28)	59.71 (15.90)	0.33
Highest education level completed: *n* (%)								0.21
Less than primary school	5598 (39.3)	3011 (61.3)	6246 (55.7)	1419 (54.0)	111 (2.6)	2172 (56.0)	1269 (27.7)	
Primary school	2791 (19.6)	607 (12.4)	1710 (15.3)	592 (22.5)	302 (7.2)	760 (19.6)	1265 (27.6)	
Secondary school	3079 (21.6)	273 (5.6)	1391 (12.4)	279 (10.6)	760 (18.1)	489 (12.6)	555 (12.1)	
High school	2001 (14.0)	839 (17.1)	1194 (10.7)	100 (3.8)	2185 (51.9)	273 (7.0)	868 (18.9)	
College/university	760 (5.3)	165 (3.4)	490 (4.4)	215 (8.2)	839 (19.9)	147 (3.8)	563 (12.3)	
Post-graduate degree	15 (0.1)	14 (0.3)	174 (1.6)	24 (0.9)	12 (0.3)	38 (1.0)	62 (1.4)	
Currently working: *n* (%)	6073 (42.8)	3524 (72.0)	4735 (42.3)	791 (30.1)	1563 (37.2)	1057 (27.5)	1360 (29.7)	0.24
Married or in partnership: *n* (%)	11915 (83.7)	2927 (60.0)	8696 (77.6)	1670 (63.5)	2397 (57.0)	2008 (52.7)	2777 (60.6)	0.26
Rural setting: *n* (%)	7319 (51.4)	2886 (58.8)	8365 (74.7)	699 (26.6)	1033 (24.5)	1275 (32.9)	625 (13.6)	0.40

* All the differences were significant at a 99% confidence level. Effect size: Cramer's *V* for χ^2^ test (categorical variables) and Cohen's *f* for ANOVA test (quantitative variables).

### Pattern of activities

Cluster analyses of activities were conducted to classify activities according to their affect patterns. A total of 43,288 activities were considered for China, 17,094 for Ghana, 41,566 for India, 4844 for Mexico, 10,560 for Russia, 13,722 for South Africa, and 25,087 for Spain. Activities (and their corresponding affective states) were collected from participants who answered sets A, B, and C. The activity “went to sleep for the night” was excluded from the cluster analyses because the affective state was not coded for this activity.

The average linkage method presented a good value for CCC in each country. CCC values ranged from 0.73 in Russia to 0.98 in South Africa. The clusters (edges in Figure S1) with high AU values (percentages higher than 95% confidence level) are strongly supported by the data. The dendrogram in Figure S1 shows the order in which the different clusters were created. Although the pattern of results was different from country to country (Figure S1), there was a tendency for leisure activities to group in one cluster, whereas household and work-related activities were in another. Some activities, such as intimate relations, were underreported, and did not show a stable pattern of association.

### Reliability and construct validity in sets A, B, C, and D

Considering the activities reported in sets A, B and C, a CFA was carried out by country to examine the construct validity and to test whether the factorial structure of affect was comprised of two factors: negative items loading on one factor and positive items loading on the other. Adequate fit indices, indicating satisfactory model fit, were found for all the countries, pooling sets A, B and C in each of them. In all cases, CFI and TLI values were higher than 0.98. RMSEA values ranged from 0.026 to 0.074. Moreover, in each group and subgroup, the upper boundaries of 90% confidence intervals were always lower than 0.080. These findings suggest that a two-factor model (negative affect and positive affect) can be considered a plausible hypothesis for the initial seven-item instrument in each country.

The results of the model shown in Table 2 are the estimates of the loadings of each observed measure on each of the factors, followed by the standard errors (s.e.) and their associated *p*-values of the null hypothesis that in the studied population the pertinent factor loading was zero. Factor correlation was negative and significant in all cases, with values ranging from −0.374 in Mexico to −0.753 in South Africa. The values associated with composite reliability in the two factors suggested an adequate reliability for the DRM in all the countries considered. Composite reliability values for negative affect ranged from 0.770 in India to 0.911 in Spain, whereas values ranging from 0.700 in Russia to 0.893 in China were found for positive affect.

**Table 2:** Confirmatory Factor Analysis of the seven DRM items (standardized factor loading estimates and their standard errors), considering activities reported in sets A, B, and C.

	China	Ghana	India	Mexico	Russia	South Africa	Spain
	(*n* = 43 288)	(*n* = 17 094)	(*n* = 41 566)	(*n* = 4844)	(*n* = 10 560)	(*n* = 13 722)	(*n* = 25 087)
**Variable**	**Loadings** * **(s.e.)**						
Negative affect							
Worry	0.929 (0.004)	0.866 (0.006)	0.771 (0.005)	0.816 (0.011)	0.867 (0.007)	0.909 (0.006)	0.907 (0.002)
Rush	0.874 (0.005)	0.740 (0.009)	0.693 (0.006)	0.813 (0.011)	0.730 (0.010)	0.779 (0.011)	0.894 (0.002)
Irritation/anger	0.949 (0.003)	0.889 (0.006)	0.839 (0.004)	0.848 (0.011)	0.864 (0.008)	0.933 (0.005)	0.939 (0.001)
Depression	0.955 (0.003)	0.917 (0.005)	0.872 (0.004)	0.857 (0.010)	0.873 (0.008)	0.951 (0.005)	0.890 (0.002)
Tense/stress	0.899 (0.005)	0.856 (0.006)	0.810 (0.004)	0.876 (0.009)	0.863 (0.006)	0.919 (0.005)	0.921 (0.002)
Positive affect							
Calm/relax	0.963 (0.005)	0.812 (0.009)	0.898 (0.005)	0.874 (0.031)	0.967 (0.011)	0.918 (0.006)	0.970 (0.004)
Enjoyment	0.921 (0.005)	0.940 (0.009)	0.942 (0.005)	0.767 (0.028)	0.704 (0.010)	0.971 (0.006)	0.812 (0.004)
Positive with negative affect	−0.613 (0.007)	−0.681 (0.010)	−0.469 (0.006)	−0.374 (0.021)	−0.742 (0.010)	−0.753 (0.010)	−0.638(0.005)
CFI	0.998	0.997	0.991	0.993	0.994	0.999	0.996
TLI	0.997	0.996	0.985	0.989	0.990	0.998	0.993
RMSEA	0.032	0.028	0.063	0.048	0.043	0.026	0.074
90% CI for RMSEA	(0.030, 0.034)	(0.024, 0.031)	(0.061, 0.066)	(0.041, 0.054)	(0.039, 0.048)	(0.020, 0.030)	(0.071,0.077)
Composite reliability							
Negative affect	0.840	0.777	0.770	0.820	0.798	0.844	0.911
Positive affect	0.893	0.745	0.850	0.701	0.700	0.843	0.800

* All the loadings were significant at a 99% confidence level.

In set D, the affect was coded for each part of the day, not separated by activity, so CFA and reliability analyses were carried out separately for each country, pooling the responses in the morning, afternoon and evening. The results in Table 3 again show a good construct validity of the affect items, divided in two factors, with adequate goodness-of-fit indices. Adequate reliability was found in each country for this version, although in some cases composite reliability for positive affect was slightly lower than 0.70. These analyses were not carried out in Spain, because set D was not used in the Spanish sample.

**Table 3:** Confirmatory Factor Analysis of the seven DRM items (standardized factor loading estimates and their standard errors) in set D, considering pooled responses corresponding to feelings in the morning, afternoon and evening.

	China	Ghana	India	Mexico	Russia	South Africa
	(*n* = 10 963)	(*n* = 3740)	(*n* = 8339)	(*n* = 888)	(*n* = 3204)	(*n* = 2890)
**Variable**	**Loadings** * **(s.e.)**					
Negative affect						
Worry	0.931 (0.005)	0.868 (0.014)	0.769 (0.010)	0.847 (0.024)	0.875 (0.010)	0.911 (0.011)
Rush	0.879 (0.007)	0.726 (0.021)	0.631 (0.012)	0.824 (0.025)	0.702 (0.015)	0.829 (0.018)
Irritation/anger	0.949 (0.004)	0.831 (0.017)	0.818 (0.008)	0.826 (0.027)	0.856 (0.011)	0.945 (0.010)
Depression	0.923 (0.006)	0.903 (0.013)	0.880 (0.007)	0.810 (0.026)	0.887 (0.011)	0.968 (0.007)
Tense/stress	0.891 (0.008)	0.871 (0.014)	0.835 (0.008)	0.820 (0.025)	0.872 (0.010)	0.846 (0.013)
Positive affect						
Calm/relax	0.936 (0.010)	0.754 (0.019)	0.916 (0.016)	0.831 (0.051)	0.953 (0.019)	0.880 (0.015)
Enjoyment	0.934 (0.009)	0.992 (0.020)	0.904 (0.016)	0.764 (0.049)	0.679 (0.017)	0.961 (0.014)
Positive with negative affect	−0.610 (0.013)	−0.685 (0.022)	−0.498 (0.012)	−0.534 (0.042)	−0.734 (0.016)	−0.734 (0.019)
CFI	0.998	0.997	0.989	0.994	0.997	0.997
TLI	0.997	0.996	0.982	0.990	0.995	0.995
RMSEA	0.036	0.025	0.072	0.042	0.035	0.040
90% CI for RMSEA	(0.032, 0.041)	(0.017, 0.033)	(0.067, 0.077)	(0.025, 0.060)	(0.027, 0.044)	(0.032, 0.050)
Composite reliability						
Negative affect	0.857	0.787	0.775	0.810	0.821	0.851
Positive affect	0.879	0.734	0.846	0.669	0.680	0.808

* All the loadings were significant at a 99% confidence level. In the sample from Spain, set D was not completed.

### Differences in affect across countries

Significant differences across countries were found for net affect, with moderately high effect size (Table 4). The highest mean net affect value was found in South Africa. All the pairwise comparisons (except Russia vs Mexico and Spain vs China) were significant, at a 99% confidence level after the Bonferroni correction, although in some cases these differences could be due to the large sample size. According to the effect size associated with these pairwise comparisons, the largest differences in net affect were found between South Africa and India (Hedges' *g* = 1.00), Mexico (Hedges' *g* = 0.92), and Russia (Hedges' *g* = 0.91). Similar results were found for the U-index, with lower values for South Africa, Spain, China, and Ghana, and higher values for India, Mexico, and Russia (see results in Tables 4 and 5).

**Table 4:** Mean scores (s.d.) of net affect and U-index, ranked from the highest to the lowest net affect.

	Net affect				U-index			
Country	Mean (s.d.)	F (6, 45651)	*p*	Cohen's *f*	Mean (s.d.)	F (6, 35779)	*p*	Cohen's *f*
		806.37	<0.001	0.33		572.27	<0.001	0.31
South Africa	91.85 (13.53)				0.10 (0.23)			
Ghana	87.42 (14.13)				0.14 (0.28)			
Spain	85.38 (14.47)				0.10 (0.24)			
China	84.54 (16.45)				0.13 (0.29)			
Russia	78.09 (16.43)				0.28 (0.37)			
Mexico	77.62 (17.78)				0.28 (0.39)			
India	75.51 (17.13)				0.33 (0.37)			

ANOVA tests comparing net affect and U-index scores among countries.

**Table 5:** Hedges' *g* associated with significant differences after Bonferroni correction in pairwise comparisons across countries in terms of net affect (lower diagonal) and U-index (upper diagonal).

	China	Ghana	India	Mexico	Russia	South Africa	Spain
China		-	0.61	0.49	0.48	0.11	0.11
Ghana	0.18		0.55	0.43	0.43	0.15	0.15
India	0.54	0.73		0.13	0.14	0.68	0.70
Mexico	0.42	0.63	0.12		-	0.59	0.61
Russia	0.39	0.61	0.15	-		0.58	0.60
South Africa	0.46	0.32	1.00	0.92	0.91		-
Spain	-	0.14	0.60	0.49	0.47	0.46	

Hedges' *g* values of 0.20, 0.50, and 0.80 constituted small, medium, and large effect sizes, respectively, according to Cohen's guidelines.

- Significant differences were not found and effect size measure was not reported.

The average number of episodes reported was 4.7 (s.d. = 1.8), ranging from 2.5 in Mexico to 6.0 in Spain; average episode duration was 85.7 minutes (s.d. = 68.9), ranging from 60.5 in India to 146.4 in Mexico.

### Diurnal variation of affect

**Figure 1:**
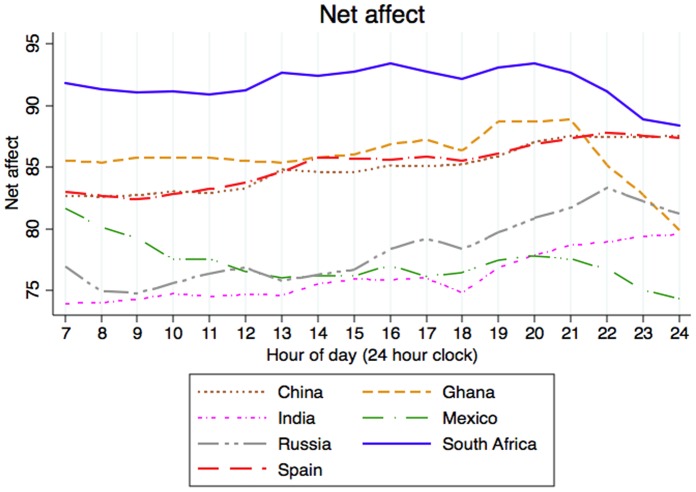
Diurnal variation of net affect in each country.

As shown in Figure 1, net affect improved as the day passed, although in some countries–South Africa, Ghana, and Russia–it declined in the evening. Mexico showed a different pattern than the other six countries, with net affect reaching its peak upon awakening and declining throughout the day.

**Figure 2:**
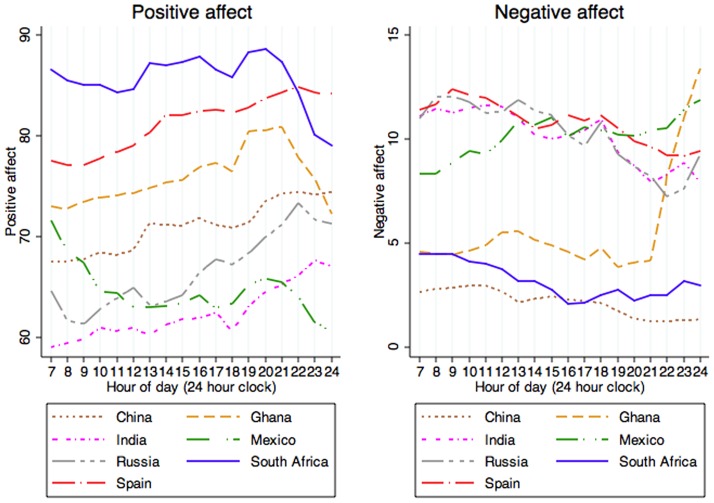
Diurnal variation of positive and negative affect in each country.

Positive affect showed a similar pattern as net affect (Figure 2). In some countries–South Africa, Spain, Russia, and Ghana–it declined early in the morning, and for all countries except Mexico it increased as the day passed, with a decline in the evening in all countries except China. In Mexico, positive affect decreased as the day passed, but increased in the afternoon and early in the evening.

Negative affect was most pronounced in the morning and tended to decrease as the day passed, although in most countries (except China) it rose in the evening. Mexico showed a different pattern, with negative affect increasing as the day passed.

## Discussion

The present study's results show that this abbreviated version of the DRM is a useful tool for multi-country studies evaluating experienced well-being. This study confirms that the abbreviated version of the DRM tested showed adequate psychometric properties regarding reliability and construct validity in the seven countries. The cluster of activities varied in each country, although the results showed a tendency to form two groups of activities: one group comprising work and household activities and therefore with a lower net affect, and another one mainly comprising leisure activities, with a higher associated net affect.

The results obtained from the fitted CFA model support the two-factor hypothesis regarding the seven affective state items of the DRM, with worried, rushed, irritated/angry, depressed, and tense/stressed loading on the negative affect factor, and calm/relaxed and enjoying loading on the positive affect factor. Lucas *et al.* [32] also found positive affect and negative affect to be discriminable from each other. Furthermore, both factors showed adequate internal consistency, although other studies using different adjectives have obtained higher rates [33].

Ghana and South Africa presented the highest net affect, whereas India showed the lowest net affect. Regarding the diurnal variation of affect, all countries showed a similar pattern, with the exception of Mexico. In general terms affect improved as the day passed (positive affect increased and negative affect declined), and in the evening it worsened (positive affect declined and negative affect increased). Previous studies in the United States with the Experience Sampling Method and the DRM have also found similar patterns regarding the diurnal rhythms of affect, with negative affect falling for most of the day [1], [34] and positive affect improving as the day passed [34].

Regarding the feasibility of use, this is a much shorter version of the DRM, since it is designed to last a maximum of 15 minutes of interview time, whereas completion times for the self-administered original DRM instrument [1] ranged from 45 to 75 minutes [1], [2]. Furthermore, the administration of the questionnaire through an interview has the advantage that it can be administered to people regardless of their level of education. The fact that more than 40% of the sample had received little or no formal education was not a barrier for the administration of the DRM. Average number of episodes reported was around five, which, as expected, is about a third of the episodes reported in the original DRM version developed by Kahneman *et al.* [1], although average episode duration was 86 minutes, higher than in the original version, which was around one hour [1].

This instrument can therefore be used to measure everyday experiences and activities and can be useful in social and health research to analyze the associations between affective states in everyday life and behavior, such as time use, or underlying biological processes [35]. Furthermore, it can be helpful for economists and policymakers to measure the well-being of the society [14].

One of the strengths of the present study is its large sample size and the fact that the recruited sample included geographically and socio-economically diverse participants who are representative of each country's population. Nevertheless, large sample sizes can mean that small differences are detected as statistically significant. For this reason, in the present study effect sizes were calculated to describe the magnitude of the differences.

The results of this study should be interpreted taking into account some limitations. Convergent validity and test-retest reliability were not assessed. However, the convergent validity of the DRM has been previously shown to be appropriate when compared with the Ecological Momentary Assessment [33], [35], and previous analyses performed with this abbreviated version of the DRM have shown a moderate temporal stability [12]. Further studies are being planned as a part of the SAGE study program to compare self-reported emotive states obtained through the DRM with ESM and other biomarkers of emotive states.

## Supporting Information

Figure S1 **Cluster dendrograms of activities in the DRM with AU/BP values.** Analyses separated for each country.
(TIFF)
