# Supplementary figures and images for: Multi-Country Evaluation of Affective Experience: Validation of an Abbreviated Version of the Day Reconstruction Method in Seven Countries
Source: PLoS One. 2013 Apr 23;8(4):e61534. doi: 10.1371/journal.pone.0061534 (PMC3634002; doi:10.1371/journal.pone.0061534)

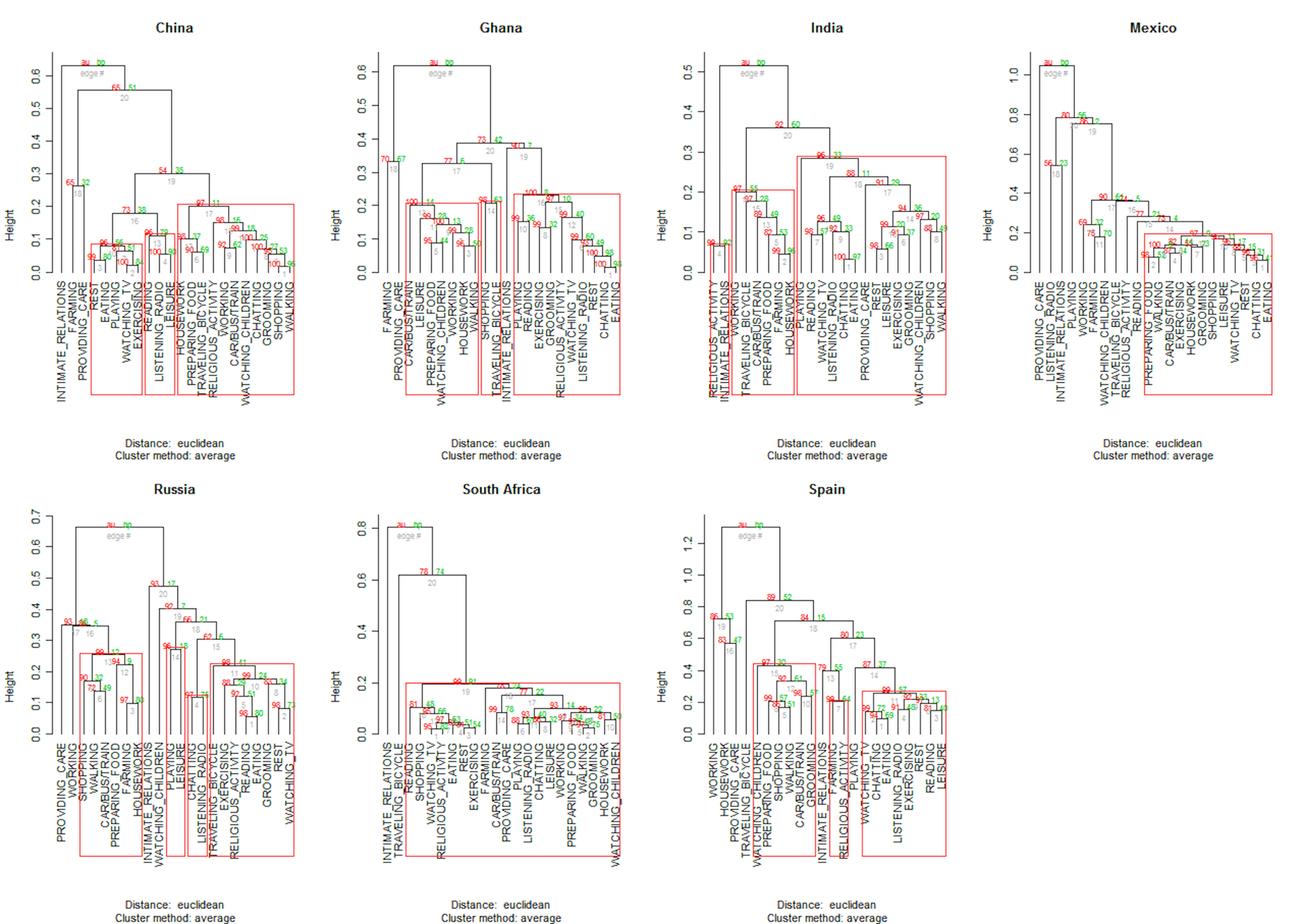

Supplement: Figure S1 — Cluster dendrograms of activities in the DRM with AU/BP values. Analyses separated for each country. (TIFF) [file pone.0061534.s001.tiff]
